# The ability of two chlorine dioxide chemistries to inactivate human papillomavirus‐contaminated endocavitary ultrasound probes and nasendoscopes

**DOI:** 10.1002/jmv.25666

**Published:** 2020-02-04

**Authors:** Craig Meyers, Janice Milici, Richard Robison

**Affiliations:** ^1^ Department of Microbiology and Immunology Pennsylvania State College of Medicine Hershey Pennsylvania; ^2^ Department of Microbiology and Molecular Biology Brigham Young University Provo Utah

**Keywords:** antiviral agents, dissemination, human papillomavirus, immune responses, papillomavirus, pathogenesis, reinfection, virus classification

## Abstract

Sexual transmission is the most common pathway for the spread of Human papillomavirus (HPV). However, the potential for iatrogenic HPV infections is also real. Even though cleared by the Food and Drug Administration and recommended by the World Federation for Ultrasound in Medicine and Biology, several disinfectants including glutaraldehyde and *o*‐phthalaldehyde have shown a lack of efficacy for inactivating HPV. Other methods such as ultraviolet C and concentrated hydrogen peroxide have been shown highly effective at inactivating infectious HPV. In this study, two chlorine dioxide systems are also shown to be highly efficacious at inactivating HPV. An important difference in these present studies is that as opposed to testing in suspension or using a carrier, we dried the infectious virus directly onto endocavitary ultrasound probes and nasendoscopes, therefore, validating a more realistic system to demonstrate disinfectant efficacy.

## Highlights

- Nonsexual transmission of Human Papillomavirus (HPV) is a real potential risk. For the first time, two chlorine dioxide chemistries are tested and shown to be efficacious in killing HPV. This study tested drying the infectious virus directly onto endocavitary ultrasound probes and nasendoscopes, therefore validating a more realistic system to demonstrate disinfectant efficacy.

## 1 INTRODUCTION

Human papillomavirus (HPV) is a small, nonenveloped DNA virus with over 200 types identified. These types are classified as either high‐risk for their implication in cancers in areas such as the cervix, uterus, and head and neck, or low‐risk types, which cause benign condylomas or warts. Types 16 and 18 are classified as high‐risk and are documented to be the most prevalent types worldwide,^1^ attributable to large numbers of cancers of the cervix, uterus, anus, and head and neck.^2^, ^3^ Sexual transmission via oral or penetrative means is widely documented in the scientific literature and is highlighted for its risk by healthcare institutions such as the National Health Service and the Centers for Disease Control and Prevention.^4^, ^5^ However, a source of potential transmission via fomites in the healthcare environment from inadequate disinfection practices has become an area of concern, debate, and discussion. Clinical areas in which examination, diagnoses, or treatment is provided through the use of instruments entering body cavities, cavities where HPV16 and 18 are prevalent, pose a risk to clinician and patient. Obstetrics, gynecology, and emergency medicine departments are examples of areas where devices including transvaginal endocavity ultrasound, colposcopes, and speculums are used to examine the cervix and can subsequently be contaminated with HPV.^6^, ^7^, ^8^, ^9^, ^10^, ^11^ Furthermore, devices such as endoscopes used within otorhinolaryngology departments are also at risk of HPV contamination.

The World Federation for Ultrasound in Medicine and Biology decontamination guidelines for transvaginal ultrasound transducers recommends disinfectants that include: 2.4% to 3.2% glutaraldehyde (GTA), *o*‐phthalaldehyde (OPA), 7.5% hydrogen peroxide, 0.5% bleach, ultraviolet C (UVC) radiation at 200 to 280 nm and chlorine dioxide.^12^ Our previous work in which testing was performed with UVC radiation at 253.7 nm, 0.525% and 0.87% bleach, and 31.5% sonicated hydrogen peroxide, has demonstrated the efficacy of these treatments in inactivating HPV16 and 18.^13^, ^14^, ^15^ Where only HPV16 was tested, disinfection was not achieved with 0.55% OPA, or 2.4% or 3.4% GTA.^13^, ^14^, ^15^ We, therefore, considered the next logical step for testing would entail the assessment of chlorine dioxide, as recommended in the guidelines noted above. These chlorine dioxide products have been referenced in otorhinolaryngology disinfection guidelines such as ENT UK^16^ and of the Official Journal of the Italian Society of Otorhinolaryngology.^17^ Published studies show the use of chlorine dioxide products across the globe in countries including the UK, Australia, New Zealand, and Singapore.^18^, ^19^, ^20^, ^21^ This report describes the testing of two chlorine dioxide products to determine their ability to adequately disinfect devices contaminated with HPV.

Here, we used a different approach for testing the two chlorine dioxide solutions against HPV16 and HPV18 vs. our previous studies, which assessed efficacy in suspension or carrier‐based assays. In this study, we contaminated actual medical devices, endocavitary ultrasound probes, and nasendoscopes, with the virus to simulate in‐use disinfection as closely as possible.

## 2 MATERIALS AND METHODS

### 2.1 Cell culture and virus production

HaCaT cells were maintained in Dulbecco's modified Eagle's medium DMEM supplemented with 10% fetal bovine serum (FBS), 0.025 mg/mL gentamicin, and 0.11 mg/mL sodium pyruvate. Primary human keratinocytes from newborn foreskin circumcision were isolated, as previously described.^22^, ^23^ The Human Subjects Protection Office of the Institutional Review Board at Penn State University College of Medicine screened our study design for exempt status according to institutional policies and the provisions of applicable federal regulations. They determined this study did not require formal IRB review because no human participants are involved as defined by federal regulations. Keratinocytes were maintained in 154 medium supplemented with a Human Keratinocyte Growth Supplement Kit (Cascade Biologics Inc, Portland, OR). Immortalized keratinocytes stably maintaining HPV episomes were cultured in E‐medium with J2‐3T3 feeder cells and grown in raft culture to produce a virus, as previously described.^22^, ^23^ Mature virus particles were harvested from tissues after 20 days.^24^, ^25^, ^26^ Rafts were harvested and the virus was isolated by homogenization in phosphate buffer (5 mM Na‐phosphate; pH 8; 2 mM MgCl_2_), as previously described.^22^, ^23^ All virus preparations for concentration and infectivity assays were treated with Benzonase (375 U) at 37°C for 1 hour to remove any unencapsidated viral genomes. Samples were adjusted to 1M NaCl and centrifuged at 4°C for 10 minutes at 10 500 rcf to remove cellular debris.

### 2.2 Virus titers

To release the viral genomes, 10 mL of a virus preparation was resuspended in 200 mL HIRT DNA extraction buffer (400 mM NaCl/10 mM Tris‐HCl, pH 7.4/10 mM EDTA, pH 8.0), with 2 mL 20 mg/mL Proteinase K, and 10 mL 10% sodium dodecyl sulfate for 2 hours at 37°C. The DNA was purified by phenol‐chloroform extraction followed by ethanol precipitation and resuspension in 20 mL TE. Titers were determined using a quantitative polymerase chain reaction (qPCR)‐based DNA encapsidation assay utilizing a Qiagen Quantitect SYBR Green PCR Kit.^23^ Amplification of the viral genome target was performed using the previously described E2 primers against a standard curve of 10‐fold serial dilutions from 10^8^ to 10^4^ copies per mL.^23^ For infection assays, HaCaT cells were seeded in 24‐well plates with 50 000 cells per well 2 days before infection. Compounds were mixed with virus and media in a total volume of 500 µL before addition to cells. An multiplicity of infection (MOI) of 10 particles per cell was used unless otherwise noted. The virus was incubated with the cells for 48 hours at 37°C and messenger RNA was harvested using a Qiagen RNAeasy Kit.

### 2.3 Instrument preparation

Instruments tested were (a) nasendoscopes and (b) endocavity ultrasound probes. An organic load (soil) of 5% FBS was added to the virus suspension and spread along the length of the insertion tube of each device, representing the part of the instrument exposed to the patient. The inoculated instruments were allowed to dry in a laminar flow cabinet for 30 minutes or until dry.

### 2.4 Disinfectants

The two chlorine dioxide disinfection procedures used were from Tristel Solutions Limited: (a) the Tristel Trio Wipes System and (b) Tristel Duo. The ability of each procedure to inactivate authentic HPV16 and 18 was evaluated separately. As a positive disinfection control, sodium hypochlorite was used at the manufacturer's recommended concentration of 0.87% (8700 parts per million) (Pure Bright Germicidal Ultra Bleach, KIK International). The use of this control was based on its previously demonstrated efficacy against HPV16 and 18, in both suspension and carrier tests.^14^, ^15^ To control for virus recovery after drying onto the probe, some probes were not treated with disinfectant and the virus was removed and tested for infectivity, as described below. All disinfectant products were used according to the manufacturer's instructions for use.

### 2.5 Disinfection procedure

The endocavity ultrasound probe and nasendoscope were disinfected using a three‐step Tristel Trio Wipes System. This included a preclean wipe to clean the instruments, a sporicidal wipe to disinfect the instrument with a contact time of 30 seconds, and a rinse wipe to remove any chemical residue. This procedure replicates the standard decontamination guidelines for semicritical medical devices, which includes a cleaning step, a disinfection step, and a rinsing step.

The second set of endocavity ultrasound probes (Siemens) was disinfected by first using a preclean wipe to replicate the removal of ultrasound gel from a sheath that would be present on a device after a clinical procedure. The device was then disinfected with two aliquots of Tristel Duo applied via a low linting Duo Wipe, utilizing a 30 second contact time for efficacy.

Nasendoscopes (Karl Storz Medical Supplies) were also used for testing and were similarly treated with Tristel Duo and the Duo Wipe, except no initial cleaning procedure, was performed. The omission of the cleaning step was to replicate a worst‐case scenario wherein the cleaning step may be missed, or if soiling remained on the device post‐cleaning.

After the procedures, a base neutralizer (7% glycine) was used to rinse and scrape 2X the chlorine dioxide treated instruments, after which they were washed 2X with phosphate‐buffered saline (PBS) to dilute any residues of chlorine dioxide left and halt further action. All samples were filtered and washed with HaCat cell media 3X and assayed for infectivity as previously described.^15^ All disinfection efficacy tests were conducted in triplicate with separate batches of the virus.

### 2.6 HPV infectivity assay

Infection was analyzed using a previously described RT‐qPCR‐based infectivity assay for E1^E4 transcript levels.^23^ The E1^E4 spliced transcript was amplified using primers specific for the spliced transcript. HPV16 and 18 infectivity assays were performed using HaCat cells, as previously described.^22^, ^23^ Complete viral inactivation was considered achieved when post disinfection infectivity assays showed equivalent or higher *C*_t_ values than uninfected controls.

## 3 RESULTS

**Figure 1:**
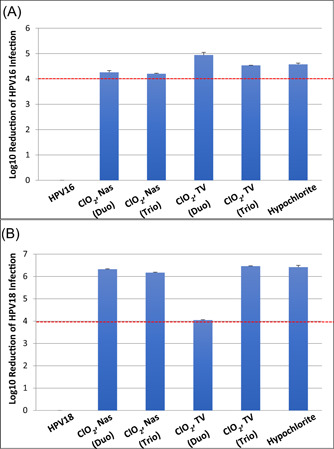
Susceptibility of HPV16 and HPV18 virions to chlorine dioxide disinfectants. A total of 1 × 10^7^ HPV16 (A) or HPV18 (B) particles were mixed with organic soil (5% FBS) and dried onto the nasendoscope (Nas) or transvaginal (TV) ultrasound probes. Two different chlorine dioxide disinfection procedures were tested; Tristel Duo (Duo) and Tristel Trio Wipes (Trio), As a control for infectious virus recovery, HPV16 and HPV18 were mixed with soil and dried onto probes, but no disinfection procedure was included. Hypochlorite was included as a positive control for disinfection efficacy. Graphs show log_10_ reduction of infectivity for each condition tested. HaCat cells were used for the infectivity assays. The dotted line marks the FDA required 4 log_10_ reductions. FDA, Food and Drug Administration

The chlorine dioxide solutions were able to produce a >99.99% reduction in infectivity of HPV16 and 18 with soil (5% BSA) included in the assays (Figure 1). The reduction is similar to that seen with 0.87% sodium hypochlorite. The differences seen in the log_10_ reduction values between the tests with the same virus type and between virus types reflect different starting titers.

The efficacy of chlorine dioxide on HPV16 was similar to that of sodium hypochlorite in our previous study, the difference is that the previous testing was performed in a suspension‐based assay, mixing the virus with the disinfectant in solution and not by applying the virus directly onto the devices, as we have done in this present study. But it did allow us to determine the differences in efficacy between different chemical groups: alcohols (ethanol, isopropanol), aldehydes (GTA, OPA), phenol and oxidizing agents (PAA‐silver, sodium hypochlorite, chlorine dioxide).^14^

## 4 DISCUSSION

In this study, we report the first results of two procedures simulating in‐use disinfection of native HPV16‐ and HPV18‐contaminated devices. These findings support our previous work, which demonstrates that oxidizing chemistries, including hydrogen peroxide, peracetic acid blended with silver, sodium hypochlorite,^13^, ^15^, ^27^ and now chlorine dioxide, are effective at inactivating both HPV16 and HPV18.

These results show that a manual procedure can be used to disinfect HPV‐contaminated devices that may not withstand methods that utilize submersion, heat, or radiation. The endocavity ultrasound probes (Siemens) and nasendoscopes (Karl Storz Medical Supplies) used for our study are representative of these devices with each device having their unique curves, ridges, and cavities that can affect the appropriate disinfection.

Furthermore and more importantly, it provides a solution to those devices that are also mobile/transportable, such as those used within the community setting by healthcare practitioners. In these scenarios, a transportable, simple method that achieves disinfection efficacy in short contact time, is sorely needed.

Medical devices for examination/diagnoses that can be used in a high patient throughput manner and be transported easily are becoming more prevalent in the healthcare industry, especially in developing countries. A good example of this is mobile colposcopy. These devices are used to examine the cervix and determine any abnormal cells or precancerous lesions that may be present. These same countries are less likely to be able to afford an automated disinfection system, and an easily transported, non‐machine‐based system for disinfection would be of great benefit.

HPV is a nonenveloped virus, which has demonstrated resistance to many disinfectants, including those which are Food and Drug Administration cleared for high‐level disinfection (GTA, OPA).^13^, ^14^, ^15^ Current guidelines require high‐level disinfection of ultrasound probes used in semicritical applications including procedures that may involve contact with mucous membranes or broken skin.^28^ By definition, high‐level disinfection refers to the complete elimination of all viruses and microorganisms, with the exception of bacterial endospores, some of which are permitted to remain.^28^

Some devices make close contact with the patient in areas in which HPV is prevalent, and studies have demonstrated that colposcopes are contaminated with HPV DNA, as are the glove boxes used by medical practitioners.^8^ Although DNA detection does not necessarily indicate the presence of viable and infective microorganisms, the work of M'Zali et al^9^ showed that HPV virions remain present on ultrasound devices used in women's healthcare, following standard disinfection protocols. This indicates that standard protocols are inadequate to properly disinfect these devices, putting both patient and clinician at risk for HPV transmission.

In addition to those devices used in women's healthcare, devices that enter the mucosal cavity of the head and neck are also at risk for contamination with HPV. In the case of emergency (eg, ambulatory) and point of use care, instruments such as those used to intubate patients with breathing difficulties, are exposed to mucosal secretions. To aid in the quick turnaround of device usage, manual disinfection procedures could be pivotal. It may also save in overall healthcare costs, as rapid disinfection methods would reduce device reprocessing downtime and also reduce the number of required devices.

A steady increase in carcinomas of the head and neck has been reported in many countries including New Zealand,^29^ Sweden,^30^, ^31^ Denmark,^32^ and the United States.^33^ Presence of HPV DNA within tumor samples has been demonstrated through PCR amplification of specific gene sections, indicative of active HPV infection. Furthermore, data demonstrate the percentage of male patients positive for HPV in the carcinomas of the head and neck is higher than that of females. It is postulated that the higher prevalence in men may be due to the higher viral load of HPV within the vagina and cervix than on the penis.^34^ Research from Hernandez et al^35^ supports these findings, revealing transmission of HPV is higher from the cervix to the penis than from the penis to the vagina. Thus, it is possible that transmission of HPV during oral sex of a man with a woman may be more likely to occur than the oral sex of a woman with a man, providing a potential explanation for the differing percentages seen. This adds another level to the importance of controlling the potential of high contamination rates on devices used in the head and neck area.
